# 3D Printing in Oral Drug Delivery: Technologies, Clinical Applications and Future Perspectives in Precision Medicine

**DOI:** 10.3390/ph18070973

**Published:** 2025-06-28

**Authors:** Zeena Saleh-Bey-Kinj, Yael Heller, Giannis Socratous, Panayiota Christodoulou

**Affiliations:** School of Medicine, European University Cyprus, 2404 Nicosia, Cyprus

**Keywords:** precision medicine, 3D printing, epilepsy, Levetiracetam, drug delivery

## Abstract

The recent advancement of 3D-printed drugs is an emerging technology that has the potential for effective and safe oral delivery of personalized treatment regimens to patients, replacing the current “one size fits all” philosophy. The objective of this literature review is to highlight the importance of 3D-printing technology in the development of personalized treatments, focusing on Levetiracetam, the first FDA-approved 3D-printed drug, for the treatment of epilepsy. Levetiracetam serves as an ideal paradigm for exploring how precision medicine and 3D printing can be applied to improve treatment outcomes for other complex diseases such as diabetes, cardiovascular diseases, and cancer. 3D printing enables precise dosage and time-release profiles by modifying factors such as shape and size, and the combination of active pharmaceutical ingredients (APIs) and excipients, ensuring consistent therapeutic levels over the treatment period. Design of oral tablets with multiple compartments allows for simultaneous treatment with multiple APIs, each one with a different release profile, minimizing drug–drug interactions and side effects. This technology also supports on-demand production, making it particularly beneficial in resource-limited or urgent situations, and offers the flexibility to customize dosage forms. Additive manufacturing could be an important tool for developing personalized treatments to address the diverse medical needs of patients with complex diseases. Therefore, there is a need for more 3D-printed FDA-approved drugs in the biopharmaceutical industry to enable personalized treatment, improved patient compliance, and precise drug release control.

## 1. Introduction

The evolution of pharmaceutical dosages started with the formulation of ointments, powders, and creams made by the ancient Greeks and Romans, from plant sources, leading to the invention of compressed tablets by Dr. Robert Fuller in 1878. The advancement of tablet production in the 20th century was driven by polymer science, leading to extended/delayed-release tablets, transdermal systems, and long-acting implants [1]. Tablet sales worldwide are projected to surpass $500 billion by the end of 2027. A challenge faced by the pharmaceutical industry in tablet production is establishing the optimum relationship between the raw material characteristics, processing parameters, and essential quality attributes [2].

Pharmaceutical practices have followed a “one-size-fits-all” system, relying heavily on standardized formulations and non-personalized dosages. Around 40% of drug delivery is orally administered. Although there have been advances in drug administration methods, the oral route is the most favored among patients because it is safe, easily accessible, affordable, convenient, and easy to use. However, it introduces essential obstacles, such as lower absorption rates and degradation during gastrointestinal transit [3]. Treating all patients with the same dose may lead to suboptimal therapeutic results due to interpatient differences in drug metabolism. This is particularly important in patients with complicated medical histories who are prescribed multiple medications, leading to drug–drug interactions. Considering the acceptability of tablets or pills among patients, there is a potential breakthrough in the personalization of oral drugs in the pharmaceutical industry. When it comes to sensitive subgroups, such as children, issues such as the lack of appropriate pediatric dosage forms, issues with drug palatability, as well as difficulty in swallowing, still exist [4]. An example is the prescription of ‘’half tablet’’ which involves destruction of the drug structure preparation, leading to altered pharmacodynamic and pharmacokinetic action of the drug, resulting in adverse effects. This is important for drugs with a narrow therapeutic index, where even a small increase in dose could be toxic or lethal [5]. This has created the need for personalized medicine, which involves treatments tailored to the individual needs of each patient.

The National Research Council has defined precision medicine as “the ability to classify individuals into subpopulations that differ in their disease susceptibility, biology, and/or prognosis, or in their response to a specific treatment. Interventions can then be concentrated on those who will benefit, sparing expense and side effects for those who will not” (National Research Council, 2011). Personalized medicine addresses the challenges posed by traditional medicine by introducing customized drug formulations, such as 3D-printed drugs, to meet patients’ needs.

## 2. Objective and Methodology

The objective of this literature review is to provide a focused analysis of 3D-printed oral drug delivery systems through the lens of precision medicine. We review the current literature on the technologies used for the production of 3D-printed drugs and discuss their potential application in personalized treatments for complex diseases. Levetiracetam (Spritam), the first FDA-approved 3D-printed drug, is used as a model to explore the potential for treating complex diseases like epilepsy, diabetes, cardiovascular conditions, and cancer. Finally, we address the challenges hindering progress in this field and discuss emerging new technologies that can revolutionize the 3D printing of drugs and pave the way for its widespread use in the clinical setting. Specifically, this review uniquely discusses 4D printing, 3D bioprinting, and the possible application of artificial intelligence (AI) in 3D printing, and critically evaluates their readiness for clinical translation.

Search was conducted using scientific databases including PubMed and Scopus, and regulatory agency reports published between 2015 and 2025. Keywords used in the search included Precision Medicine; 3D Printing; Epilepsy; Levetiracetam; and Drug Delivery. Articles not available in English, lacking full text, or unrelated to human oral drug delivery were excluded.

## 3. 3D Printing for Drug Delivery

### 3.1. 3D Printing

3D printing, also referred to as additive manufacturing, is a transformative technology that creates objects layer by layer, utilizing a variety of materials. It originates back to the 1980s when Chuck Hull invented stereolithography, the first 3D-printing technique that solidified liquid resin utilizing UV light. Initially used for speedy prototyping in engineering and construction, 3D printing has since expanded into different fields, such as health services, fabrication, and aerospace. 3D printing also has important applications in medical research, such as the use of bioprinting technology in the development of organoids [6,7]. More recently, 3D printing has emerged in the pharmaceutical field, with the first FDA-approved 3D-printed drug, Levetiracetam (LEV), introduced in 2015, a significant milestone in drug delivery systems [8]. 3D printing enables the fabrication of multilayered dosage forms or a combination of drugs to treat multiple pathologies of specific patients using a single dose. The benefit of 3D-printed dosage forms lies in their combination of many layers, each engineered for creating profiles that facilitate both sequential and sustained drug delivery. This capacity allows for better precision in regulating the timing and localization of drug release, leading to improved therapeutic results [9]. Computer-aided design (CAD) software enhance the printing process, allowing for the exact manufacturing of drug dosage forms with adjustable attributes like shape, size, drug release properties, and blends of active pharmaceutical ingredients (APIs) customized for individual patient requirements. Combining many APIs into a single pill, known as ‘polypill’, will allow its use in specific, susceptible subgroups, enhancing patient adherence [10,11]. The geometry of 3D-printed drugs could be engineered to enable the gradual release of the active substances, thereby following consistent plasma drug levels, and reducing dosing frequency [1]. The properties of 3D-printed drugs are also determined by parameters such as porosity and infill density. Porous formulations show rapid disintegration, and therefore fast drug release, while formulations with greater infill densities exhibit slower drug release due to a smaller surface area and porosity [12,13]. By optimizing pharmacokinetics, 3D-printed formulations remarkably enhance drug absorption and bioavailability, ensuring that the active components are delivered efficiently to the target sites [14]. More specifically, with 3D printing, the distribution of the drug to the cells and extracellular matrix can be precisely controlled.

One of the most impactful applications of 3D printing in oral drug delivery is in addressing the unique needs of pediatric and geriatric populations. Children often struggle with swallowing conventional tablets and capsules, and there is a lack of commercially available children’s dosage forms [4]. Similarly, geriatric patients frequently experience dysphagia and use multiple drugs with different pharmacokinetic profiles, making standard dosage forms suboptimal or even unsafe [15]. 3D printing of rapidly disintegrating porous tablets and chewable, or orodispersible films, reduces swallowing difficulties. This allows the production of customized and palatable formulations for geriatric and pediatric patients [12,16]. It also enables precise dose titration and personalization, avoiding the risks associated with tablet splitting or crushing, particularly in drugs with a narrow therapeutic index [5].

Despite the advantages, the use of 3D printing in pharmaceutical development has led to new and growing challenges. An important issue is the production of 3D-printed drugs in the clinical setting, such as in hospitals and pharmacies. A major hindering factor in the production of 3D-printed oral formulations is the lack of materials, such as pharmaceutical-grade polymers, specialized solvents and binders, and excipients, with the desired specifications and stability [17,18]. There is also limited data on the biocompatibility and stability of printable materials, which may compromise safety [19]. This is particularly important for pediatric formulations as there are insufficient stability and safety data for novel dosage forms such as gummies or orodispersible films [20,21]. Continuous modifications to printer parameters such as infill density and layer thickness, and material properties, can influence drug properties, making the development of reproducible manufacturing protocols particularly difficult. For this reason, 3D-printed formulations require continued real-time validation. However, current regulations are designed for the large-scale production of conventional pharmaceutical formulations, which are carried out by retrospective batch-based assessments. Therefore, these regulations are difficult to apply to small-batch production of 3D-printed drugs in decentralized clinical sites. Currently, regulatory agencies such as the FDA and EMA do not have specific guidelines for 3D printing, introducing further obstacles to the commercialization of these products [22]. Limited availability and high costs of equipment and material, in combination with the slow rate of production, are also factors hindering the large-scale production of 3D-printed pharmaceuticals. Slow production rates of 3D-printed drugs are due to the multistep, layer-by-layer procedures employed by most printing techniques. Finally, the specialized equipment used requires high levels of expertise, which is difficult to find in clinical settings [12].

### 3.2. 3D-Printing Techniques

There are a number of techniques currently used for the 3D printing of drugs. The choice of method used is determined by several factors such as the type and stability of the APIs and excipients, the quantity of the product required, and the available time and budget [23]. The optimal choice of 3D-printing technique ensures improved cost-effectiveness and quality (Table 1).

Stereolithography (SLA) utilizes a photopolymer resin, a liquid material composed of monomers and oligomers, that hardens when exposed to a UV laser, to create solid layers. Each layer is selectively polymerized before the object is lowered into the resin for the next layer [24]. SLA has several benefits, such as high-resolution structures created with complicated details, ideal for producing complex drug delivery systems. Furthermore, SLA is cost-and time-effective and allows for controlled integrity of materials. However, it is limited to photopolymer materials, which may not be suitable for all drug formulations, and in some cases may have safety issues [25]. Most photopolymers and UV-curable resins used in SLA are not biocompatible and show toxicity. Therefore, they cannot be used for the production of pharmaceuticals. This introduces regulatory concerns as the safety profiles of many materials are not suitable for SLA-printed dosage forms [19].

Selective laser sintering (SLS) utilizes a laser to sinter powdered material, fusing it layer-by-layer; the layers are held together by strong, stable bonds to create a solid structure. This process eliminates the need for other supportive structures, as unsolidified powder surrounding the model provides support through the procedure and might be easily removed as the process is finished. SLS creates porous tablets, which enables the development of formulations with customized drug release patterns. This technique also does not require pre-processing of material or inclusion of additional excipients that could pose potential toxicity to the formulation, therefore enhancing safety [26,27]. As this printing technique does not require the use of solvents, it provides better stability to drug substances that can undergo hydrolysis. SLS can also improve the solubility and bioavailability of poorly water-soluble drugs, such as carvedilol, by partially converting the crystalline form of the drug to an amorphous form [28]. However, it is limited by the potential degradation of heat-sensitive drugs and the lack of approved pharmaceutical-grade materials compatible with this process [18]. Most commercially available materials are industrial-grade polyamides, polystyrenes, and polycarbonates, which are unsuitable for oral drug delivery as they have limited biocompatibility and safety testing [19].

Fused deposition modeling (FDM) uses a heated nozzle, which softens and extrudes a thermoplastic filament, depositing it layer-by-layer to generate the desired shape. FDM is cost-effective, accessible, and does not require the use of non-volatile materials [29]. Another advantage is the low percentage of friability of FDM-generated tablets, which alleviates the need for hardness tests [30,31]. However, it is often limited by material compatibility, the potential for thermal degradation of APIs, and a reliance on pre-manufactured filaments [8,32].

Direct powder extrusion (DPE), is a single step process in which a powder blend is directly added to the printhead and fed towards a heated nozzle by a single screw, extruding drug-loaded polymer filaments to print the designed objects in a layer-by-layer fashion. This strategy can be used with less manufacturing expertise, and it can simplify 3D printing by transforming the two-step process of FDM into a time-effective single-step process. Therefore, it has great value in the clinical setting, such as the production of oral tablets in hospital pharmacies [33]. DPE also requires only a few grams of the powder blend and is therefore cost-efficient. Limitations of this 3D-printing technique are the difficulty in selecting compatible excipients and APIs, the potential for thermal degradation of active ingredients, and a reliance on pre-manufactured filaments [8,32,34].

Binder jet printing (BJP) deposits a liquid binding agent onto a powder bed, layer-by-layer, forming solid structures through agglomeration, a process of adhering molecules together. This procedure does not require heat and can be used for producing highly porous tablets, in various geometric forms, enabling rapid drug release and compatibility with a wide range of materials. However, the resulting tablets may have lower mechanical strength due to the agglomeration of loose powder particles by the binder agent, resulting in printed structures that lack a dense, tightly bonded matrix [35]. It is also not suitable for sustained release formulations as the porosity enables rapid disintegration, limiting the ability to control drug release over extended periods of time [19,36].

Semi-solid extrusion (SSE), also known as pressure-assisted microsyringe extrusion (PAM), extrudes a semi-solid material, such as a gel or paste, through a nozzle to create layers. These layers solidify upon cooling or solvent evaporation. It enables formulation without the application of high heat and supports bioprinting applications. However, it needs post-printing processes such as drying, which leads to challenges like shape breakdown and solution retention. Also, SSE requires high temperatures, making it unsuitable for thermolabile drugs [37].

Inkjet printing is a recent development in 3D printing that involves drop-by-drop deposition of liquid through a nozzle. This method enables accurate dosing and controlled drug release, ideal for personalized multi-drug formulations. It is suitable for the production of orodispersible films, allowing efficient absorption of the drug through the oral mucosa. Drawbacks of this method include the selection of carrier fluids to ensure consistent droplet formation and the prevention of nozzle obstruction [38]. Another consideration is the viscosity of the formulations and problems with API adsorption and material leaching, which can affect drug distribution and cause equipment failure [16].

There are also a number of more recently developed 3D-printing techniques used for drug production, such as UV curable inkjet 3D printing, Continuous Liquid Interface Production (CLIP), Melt-Electrowriting (MEW) Technology, and Electrohydrodynamic Jet (E-Jet) [39,40,41,42].

A recent development in 3D-printing technologies is deep eutectic solvents (DES), a novel class of green solvents that are composed of hydrogen bond acceptors and donors. The eutectic mixture has a lower melting point than the individual components. DES are biocompatible, customizable ink systems that can improve the solubility of poorly water-soluble drugs without the need for additional solvents. DES can be used for BJP and SSE printing due to its reduced toxicity and adjustable viscosity. The unique properties of DES make them suitable as ink materials for printing drugs with thermolabile APIs, as they do not require very high temperatures. Therefore, DES are ideal for 3D printing of drugs with precise dosing and drug-release profiles, required for precision medicine [43,44].

The technique of 3D printing for drug delivery necessitates careful data collection and analysis to ensure both accuracy and efficacy. Data regarding characteristics such as APIs, excipients, and specific drug release patterns is collected and examined by software prior to the printing stage. Advanced tools assess these traits, ensuring consistency with 3D-printing methods. CAD software is used to create detailed digital blueprints that guide the 3D-printing process [45]. Therefore, it enables the customization of dosage forms, such as particular shapes, dimensions, and inner geometries, that can impact drug release profiles. Following CAD, computer-aided manufacturing (CAM) translates these designs into machine-readable instructions, guaranteeing precise layer-by-layer production. Together, CAD and CAM enable the fabrication of highly accurate and personalized drug delivery systems [46].

## 4. 3D-Printed Drugs in Epilepsy

3D-printed drugs have the potential to significantly improve the management of complex diseases that require a personalized treatment approach. Epilepsy is a chronic neurological disorder characterized by abnormal electrical activity in the brain, causing repeated seizures, which entail heterogeneity in its clinical manifestations [47]. Treatment strategies include anti-epileptic drugs, lifestyle modification, and, in some cases, surgery. The mainstay treatment for epilepsy is fixed-dose anti-seizure medications (ASMs).

ASMs reduce the frequency and intensity of seizures by decreasing electrical activity in the brain. More specifically, they prevent neuronal depolarization by blocking sodium or calcium channels, enhancing potassium channel activity, inhibiting glutamate-induced neuronal excitation, or promoting GABA-induced neuronal inhibition. There are three generations of ASMs, based on the time of their development [48]. Treatment choice depends on factors such as seizure type and frequency, severity, underlying cause, age, gender, tolerability, and comorbidities. Only up to 70% of newly diagnosed patients living with epilepsy can be treated successfully [47].

Current epilepsy therapies have reduced efficacy due to disease heterogeneity [49]. Heterogeneity in epilepsy arises from various etiologies, such as genetic mutations, brain trauma, and metabolic disorders, and presents significant challenges in the development of targeted and effective therapies [50]. Therefore, a lack of personalization in the treatment of epileptic patients frequently results in suboptimal seizure elimination, unwanted adverse effects, and decreased patient adherence. Additionally, drug efficacy is restricted by the blood–brain barrier (BBB), which limits therapeutic agent delivery to the brain [51].

The International League Against Epilepsy categorizes epilepsy into four primary types, emphasizing its heterogeneity: (i) focal epilepsy where seizures arise from a distinct region within a single cerebral hemisphere, (ii) generalized epilepsy where seizures affect both sides of the brain at the same time, (iii) combined focal and generalized epilepsy, where patients have both focal and generalized seizure experiences, and (iv) unknown epilepsy where the cause of seizures is ambiguous or cannot be established [52]. This means that each patient needs to be treated with specific drugs or a combination of drugs, at specific dosages and frequencies.

LEV is a second-generation ASM approved as a supplementary therapy for seizure disorders. Moreover, LEV is gaining significance in precision medicine for epilepsy because of its low drug interactions, reliable pharmacokinetics, and extensive therapeutic index, positioning it as a suitable option for personalized treatment strategies [53]. LEV’s distinctive mechanism of action, involving the modulation of synaptic vesicle protein 2A, enables a more precise therapeutic strategy, potentially tackling these individual differences more efficiently than conventional anti-seizure drugs. Moreover, LEV exhibits better BBB permeability than other anti-seizure medications, improving its effectiveness and decreasing the necessity for higher doses that may cause side effects [54].

3D-printed drugs could enable personalized treatment of epileptic patients. A pharmaceutical firm, Aprecia Pharmaceuticals, used a patented BJP platform termed ZipDose technology for the commercial, large-scale production of 3D-printed tablets, named Spritam [22]. Spritam received approval by the US Food and Drug Administration (FDA) on August 3, 2015. The printing method involves the spreading of drug particles into thin layers, proceeding with selective jetting that binds the particles into thin, porous layers. This method gives the 3D-printed LEV tablet a highly porous internal environment with micron-scale pore size that increases surface area. The effectiveness of Spritam was determined to be comparable to that of the standard traditional pressed tablet. Nonetheless, it has a lower solubilization time because of its porous and soluble matrix, leading to ultra-rapid drug dissolution and faster onset of action [17,55]. Spritam, designed to dissolve rapidly in liquids, was created to assist patients with dysphagia and children who struggle with swallowing large pills [56]. Consequently, Spritam is the first fast-acting, immediate-release dosage form of LEV, which breaks down and completely disperses in under five seconds despite having a high active drug dosage (1000 mg) [57,58]. Conversely, standard LEV breaks down in under 60 s. The favorable pharmacokinetics of LEV, along with the unique delivery method of 3D-printed Spritam, help enhance personalized treatment approaches for epilepsy [59].

This technology can be further improved to facilitate the personalized treatment of epileptic patients [36,60]. More specifically, 3D-printed tablets with multiple compartments would allow treatment with multiple medications or different doses of the same medication. This enables accurate adjustment of medication dosages according to specific patient requirements, enhancing effectiveness and reducing side effects [61]. By adjusting the wall thickness and excipient composition of each compartment, sequential release of the content of each compartment, at specific time intervals, can be achieved. This would mean that although the patient will take a tablet once a day, each medication or dose will be released at a different time during the day [62].

## 5. Application of 3D-Printed Drugs in Other Diseases

The current advances in the development of 3D-printed medications, including the FDA-approved LEV for epilepsy, showcase this technology’s potential to transform treatment for various other illnesses. In diabetes management, 3D printing can facilitate the creation of tailored tablets that release glucose-regulating medication based on the patient’s unique metabolic profile. This reduces the likelihood of under- or overdosing that could result in hypo- or hyperglycemia, respectively [63]. Additionally, research has examined the application of 3D-printing technology in other diabetes treatments. For instance, glimepiride and metformin, two antidiabetic drugs with varying daily dosing regimens, were merged to form a bilayer formulation. Dissolution studies indicated that each medication was released within the intended timeframes, with glimepiride released within 75 min and metformin within 480 min [64,65]. Moreover, a different study reported the creation of 3D-printed tablets containing metformin HCl–loaded polyvinyl alcohol (PVA) through a refined aqueous solvent diffusion method [66]. The creation of 3D-printed gummies containing metformin designed for children illustrates the potential of this technology for producing patient-friendly dosage forms that improve adherence and facilitate accurate control of drug release profiles. Specifically, this formulation ensures metformin is not released in the oral cavity, leading to better palatability [21]. Considering the diversity among diabetic patients, where elements like age, genetic factors, lifestyle choices, and existing health conditions affect treatment outcomes, 3D-printed formulations tailored to unique patient requirements can optimize symptom control [67]. Another study reports the design of a DuoTablet, which entails a tablet embedded within a larger tablet, allowing the release of glipizide at two different times, due to a difference in dissolution of the two compartments. This design reduces the number of doses a patient takes daily, increasing compliance [68]. Personalized oral delivery systems could combine antidiabetic medications, with different release rates and dosages, into one pill, enhancing glycemic management and minimizing side effects. This method improves compliance, reduces the chance of treatment failure, and ultimately results in improved long-term health outcomes [69].

Patients with cardiovascular disease often have a multitude of conditions such as hypertension, dyslipidemia, arrhythmias, and heart failure, and, therefore, receive different medications, at different doses and times. 3D-printed polypills that merge antihypertensive drugs, statins, and anticoagulants could significantly enhance patient compliance and clinical results. Khaled et al. designed a five-in-one dose combination polypill with defined immediate and sustained release profiles of atenolol, pravastatin, ramipril, aspirin, and hydrochlorothiazide for the treatment of metabolic syndrome [70]. Another multilayer tablet containing lisinopril, indapamide, rosuvastatin, and amlodipine has been reported as a potential treatment for hypertension and dyslipidemia. This formulation would enable simultaneously taking multiple drugs at tailored doses, or multiple doses of the same drug at different times, increasing clinical efficacy and limiting drug–drug interactions [71]. Another study demonstrates a successful 3D extrusion printing of a multi-compartment tablet that delivers three drugs through two different release mechanisms: diffusion through gel layers and osmotic release through a controlled porosity shell. This results in captopril exhibiting a zero-order release, and glipizide and nifedipine showing sustained release kinetics dependent on the API/excipient ratio [13]. 3D printing can therefore enable the development of personalized or unique formulations and more complex drug-release profiles that align with circadian cycles, like morning spikes in blood pressure or coagulation processes.

In cancer treatment, 3D-printed tablets can merge various chemotherapeutic drugs into one formula intended for regulated or sequential release, enhancing tumor targeting and reducing toxicity and adverse effects [72,73]. Another approach is using 3D-printed porous absorbers, which can absorb residual chemotherapeutic agents from the blood, therefore reducing systemic toxicity to healthy tissues [40]. 3D-printed implantable scaffolds are an alternative approach to oral administration of chemotherapeutics. These drug delivery systems allow prolonged and sustained drug release for periods of up to several months, avoiding the need for hospitalization during chemotherapy. Furthermore, they limit systemic adverse effects, as they deliver the drug directly to the target area. This approach has been extensively covered in other reviews [41,42,74,75].

These applications show how 3D printing can be tailored to meet individual patient requirements for different illnesses, indicating a future where personalized medicine is common in healthcare. The above studies, as well as additional examples of oral 3D-printed drugs under preclinical development, are summarized in Table 2.

## 6. Emerging Technologies for 3D-Printed Drugs

Ongoing research is exploring 3D printing for novel drugs and treatments. The Chinese pharmaceutical company Triastek (Nanjing, China) developed the drugs T19, T20, and T21 using a patented 3D-printing platform known as MELT^®^ (Melt Extrusion Deposition). This technology is a specialized type of FDM, which offers more precise control of the geometry and distribution of both APIs and excipients. This allows the design of multi-compartment drugs with complex release profiles, enabling the large-scale production of personalized drug formulations [81,82]. T19 is used for the treatment of rheumatoid arthritis through a timed-release approach that aligns with the body’s natural pain rhythms. T20 is a treatment for cardiovascular diseases, whereas T21 is a specific treatment for ulcerative colitis, designed to administer the medication directly to the colon [67,83]. Additionally, studies are underway to create 3D-printed drugs for addressing childhood cancer, emphasizing the importance of accurate dosages and an appealing taste. Studies are investigating 3D-printed vaccines and biologics, presenting a potential enhancement for global healthcare access [20].

**Figure 1:**
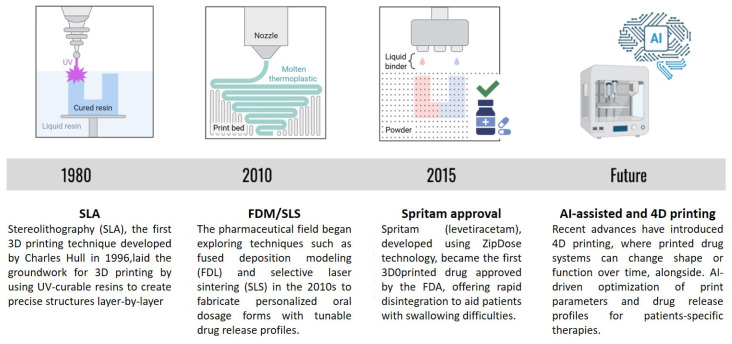
**The evolution from 3D to 4D and AI-driven drug printing**. Progression from traditional 3D-printing towards 4D-printing and AI-integrated technologies, highlighting their increasing potential for personalized drug delivery. Parts of the figure were created using Biorender.

Four-dimensional (4D) printing is a recent innovation in the field, which is still under intensive research. 4D printing for drugs refers to the use of 3D-printing technology to create drug delivery systems that can change their shape, structure, or properties over time in response to environmental stimuli. In this context, the “fourth dimension” is time, as the printed structures are designed to evolve or transform after printing, to improve drug release or targeting (Figure 1). For example, a 4D-printed drug delivery system might be designed to respond to pH changes in the body, such as releasing drugs in specific areas of the gastrointestinal tract, where the pH is appropriate. Alternatively, drugs may expand in response to temperature or moisture, allowing the release of the drug in a controlled manner over time [84].

Looking to the future, the integration of 3D bioprinting with neurological treatments holds enormous capabilities and potential for therapy. Patient-specific neural implants, biosensors for real-time feedback, and responsive rehabilitation systems could fundamentally improve the management of epilepsy and other complex diseases. These technologies promise to offer more accurate, adaptive, and treatment-oriented interventions focusing on each patient’s needs. Despite the difficulties when it comes to scalability, regulations, and development expenses, 3D printing serves as a transformative tool in addressing unmet needs in disease treatment, paving the way for further focus on personalized patient care solutions.

The application of AI has the potential to transform 3D drug design and manufacturing. The ability of AI algorithms to analyze large datasets of drug properties, patient clinical characteristics, and printing parameters, may enable optimization of formulation design and drug release profiles, enhancing precision, consistency, and personalization. AI also enables the screening of the compatibility and stability of excipients and APIs in various printing techniques. Therefore, by combining AI with 3D printing, we can move closer to achieving on-demand, tailored treatments with improved speed, safety, and scalability (Figure 1) [85,86].

## 7. Conclusions

3D printing provides a revolutionary approach by enabling the creation of highly customized drug formulations. It allows accurate dosage, customized drug release patterns, and the capability to merge various active components into a single tablet. This reduces the frequency of drug administration and improves patient compliance [87,88]. This technology additionally facilitates on-demand production, rendering it especially advantageous in situations with limited resources or urgent needs.

In epilepsy and other complex diseases like diabetes, cardiovascular disease, metabolic syndrome, and cancer, these sophisticated delivery systems improve the transportation of active substances to their site of action and increase the therapeutic efficacy. Additionally, 3D printing enables the integration of various APIs into a single formulation, permitting a blend of therapies that can tackle multiple pathways. These advancements are essential for handling the clinical characteristics of the condition. Additionally, customizing dosage form and formulation, by altering taste, shape, and size, makes medications attractive to patients who are hesitant to follow their treatment. Personalized medications enhance patient adherence, particularly for groups struggling with swallowing or those on medications with limited therapeutic windows.

Despite its potential, the technology faces significant drawbacks. Its slow production speeds make it unsuitable for mass production. The high cost of 3D printers, coupled with the high cost of materials and the need to adhere to regulatory requirements, contributes to their limited acceptance. Additionally, worries persist about the quality control and reproducibility of printed medications, especially in decentralized manufacturing environments such as pharmacies. To approach these challenges, progress in materials science, printing technology, and regulatory systems is essential [89].

Health Technology Assessment (HTA) is a process that looks at the medical, economic, social, and ethical aspects of health technologies. It is an important framework for adding 3D-printed drugs to healthcare systems. Integrating 3D-printing technologies into health care systems is especially important, particularly for rural areas, which have limited access to medical services. Although implementation involves high upfront costs, including equipment, materials, and training, these may be offset over time by simplifying polypharmacy, improving adherence, reducing hospitalizations, and minimizing drug waste through personalized dosing [90]. Easier administration of drugs and fewer side effects may lead to better outcomes for patients, but ethical issues about fair access exist. Future HTA models should be tailored to the specificities of additive manufacturing and supported by real-world data and pilot studies to inform sustainable adoption [12,18].

Recent advances in this field could potentially revolutionize the 3D printing of oral drugs for complex diseases. An example is 4D printing, which allows controlled release of drugs according to physiological conditions. Also, in the future, combining 3D printing with AI and bioprinting could lead to more personalized and effective treatments (Figure 1).

## Figures and Tables

**Table 1:** Advantages and disadvantages of 3D-printing techniques.

Technique	Advantages	Limitations	References
Stereolithography (SLA)	High-resolution, complex delivery systems, cost-effective, and material integrity control	Limited to photopolymer materials, potential material safety concerns, and regulatory concerns	[19,24,25]
Selective Laser Sintering (SLS)	Porous structures, no need for solvent or additional excipients, customizable release, no need for pre-processing	Not suitable for heat-sensitive drugs, limited pharmaceutical-grade materials	[18,19,26,27,28]
Fused Deposition Modelling (FDM)	Cost-effective, accessible, use of non-volatile materials, low percentage of friability—no need for hardness tests	Thermal degradation risk, limited drug–polymer combinations, require preformed filaments	[8,29,30,31,32]
Direct Powder Extrusion (DPE)	Single-step, cost and time-efficient, low material quantity needed, no need for solvent	Limited excipient/API compatibility, thermal degradation of APIs, reliance on pre-manufactured filaments	[8,32,33,34]
Binder Jet Printing (BJP)	No heat needed, highly porous tablets, various geometric forms created, scalable with FDA precedent (Spritam)	Lower mechanical strength, not ideal for extended-release forms	[19,35,36]
Semi-Solid Extrusion/Pressure-assisted microsyringe extrusion (SSE/PAM)	Suitable for different formulation types (paste, gels, or suspensions), suitable for immediate-release forms	Requires post-processing (drying), poor mechanical integrity- risk of shape distortion and solution retention, unsuitable for thermolabile drugs	[37]
Inkjet printing	Drop-by-drop deposition—accurate dosing, controlled drug release, improved oral mucosal absorption	Carrier fluid selection—nozzle obstruction, viscosity limitations, risk of API adsorption, and material leaching	[16,38]

**Table 2:** Overview of oral 3D-printed drug formulations under preclinical development, categorized by APIs, dosage form, printing technique, material/excipients used, and disease application.

APIs	Dosage Form	Printing Method	Material/Excipients	Disease	Reference
Metformin; Glimepiride	Bilayer tablet	Fused Deposition Modelling	Eudragit^®^ RL PO; Mowiol^®^ 4–88 (Polyvinyl alcohol; PVA)	Diabetes	[64]
Metformin	Tablet	Fused Deposition Modelling	Polyvinyl alcohol (PVA)	Diabetes	[66]
Metformin	Gummies	Semi-solid extrusion	Starch; gelatine	Diabetes	[21]
Dapagliflozin	Tablet/Paste	Semi-solid pressure-assisted microsyringe (PAM) extrusion-based 3D printing	Caproyl 90; octanoic acid; polyethylene glycol (PEG) 400; poloxamer 188; PEG 6000	Diabetes	[76]
Glipizide	DuoTablet (tablet embedded within a larger tablet)	Fused deposition modelling	Polyvinyl alcohol (PVA)	Diabetes	[68]
Aspirin; Hydrochlorothiazide; Pravastatin; Atenolol; Ramipril	Multilayer tablet	Extrusion-based 3D printing	Cellulose acetate; d-mannitol; polyethylene glycol (PEG 6000)	Hypertension; Dyslipidemia	[70]
Captopril; Nifedipine; Glipizide	Multilayer tablet	Extrusion-based 3D printing	Cellulose acetate; d-mannitol; polyethylene glycol (PEG 6000)	Hypertension; Diabetes	[13]
Enalapril; Hydrochlorothiazide	Bilayer tablet	Fused Deposition Modelling	Triethyl citrate; Tri-calcium phosphate; Eudragit EPO	Hypertension	[77]
Lisinopril; Spironolactone	Multilayer tablet	Binder jetting 3D printing	Hyaluronic acid; polyethylene glycol (PEG)	Hypertension	[78]
Carvedilol	Tablet	UV curable inkjet 3D printing	Irgacure 2959; photocurable N-vinyl-2-pyrrolidone (NVP); polyethylene glycol (PEG) diacrylate	Hypertension	[39]
Amlodipine; Lisinopril	Multilayer tablet	Stereolithography	Candurin^®^ Gold Sheen; Polyethylene oxide	Hypertension	[79]
Lisinopril; Indapamide; Rosuvastatin; Amlodipine	Multilayer tablet	Fused deposition modelling	Polyvinyl alcohol (PVA)	Hypertension; dyslipidaemia	[71]
5-fluorouracil	Tablet	Binder jet printing	Soluplus^®^ (SOL); polyethylene glycol (PEG)	Cancer	[73]
5-fluorouracil; Cisplatin	Bilayer tablet	Pressure-assisted microsyringe	Poly (lactic-co-glycolic acid) (PLGA); Triethyl citrate	Liver cancer	[72]
Doxorubicin	Absorber	Continuous Liquid Interface Production (CLIP)	Polystyrenesulfonate	Cancer	[40]
Paclitaxel; Rapamycin; Lidocaine	Multilayer tablet	Extrusion-based 3D printing	Poly-lactic-co-glycolic acid (PLGA)	Cancer	[80]

## Abbreviations

The following abbreviations are used in this manuscript:

APIs	Active pharmaceutical ingredients
ASMs	Anti-seizure medications
BBB	Blood–brain barrier
BJP	Binder jet printing
CAD	Computer-aided design
CAM	Computer-aided manufacturing
DES	Deep eutectic solvents
DPE	Direct powder extrusion
FDM	Fused deposition modeling
HTA	Health technology assessment
LEV	Levetiracetam
PAM	Pressure-assisted microsyringe extrusion
PVA	Polyvinyl alcohol
SLA	Stereolithography
SLS	Selective laser sintering
SSE	Semi-solid extrusion
